# Unlocking Lung Cancer Cell Dormancy: An Epigenetic Perspective

**DOI:** 10.3390/ijms262210997

**Published:** 2025-11-13

**Authors:** Federico Pio Fabrizio

**Affiliations:** Department of Medicine and Surgery, “Kore” University of Enna, 94100 Enna, Italy; federicopio.fabrizio@unikore.it

**Keywords:** lung cancer, tumor dormancy, epigenetics, DNA methylation, therapy resistance, minimal residual disease

## Abstract

Lung cancer remains one of the leading causes of cancer-related mortality worldwide, with tumor recurrence and metastasis posing significant challenges despite advances in targeted therapies and immunotherapy. Cellular dormancy, a reversible, quiescent state marked by cell cycle arrest, has emerged as a key driver of therapeutic resistance and disease relapse, particularly in small-cell lung cancer (SCLC) and non-small cell lung cancer (NSCLC). Multiple mechanisms, including autophagy, stress-adaptive signaling, microenvironmental cues, and epigenetic dysregulation, have been implicated in the regulation of dormancy and long-term cell survival. Among these, epigenetic modifications such as DNA methylation, histone modifications, and non-coding RNAs (ncRNAs) play pivotal roles in maintaining dormancy by repressing proliferative gene expression programs. Increasing evidence suggests that dormant tumor cells harbor distinct epigenomic signatures, which may serve as predictive biomarkers for minimal residual disease (MRD) and relapse risk. This review summarizes current advances in understanding the epigenetic regulation of cellular dormancy in lung cancer, with a particular emphasis on the interplay between epigenetic modifiers and oncogenic signaling pathways. Furthermore, emerging molecular targets and associated therapeutic agents currently under clinical evaluation are presented, emphasizing how a deeper understanding of the epigenetic landscape governing dormancy may inform the development of novel interventions to improve long-term clinical outcomes in lung cancer patients.

## 1. Introduction

Cancer dormancy is an essential yet often underestimated phase in tumor progression, characterized by a temporary cessation of proliferation that allows tumor cells to evade therapy and immune-mediated elimination [1]. Dormancy can manifest as tumor mass dormancy, where cell proliferation is balanced by apoptosis, often due to immune pressure or restricted angiogenesis, or as cellular dormancy, wherein individual disseminated tumor cells (DTCs) enter a reversible quiescent state while remaining viable and potentially metastatic [2].

Dormant cancer cells represent a major clinical challenge due to their intrinsic resistance to cytotoxic therapies and their role in late relapse [3]. A growing body of evidence indicates that autophagy is a key survival mechanism during dormancy, enabling cells to recycle intracellular components and adapt to metabolic stress [4,5]. Concurrently, immune escape mechanisms, including reduced antigen presentation and upregulation of immune checkpoint molecules, help dormant cells persist in immunocompetent hosts. Notably, dormancy is regulated by both extrinsic microenvironmental cues and intrinsic molecular programs, including genetic and epigenetic alterations [6].

While somatic mutations can enhance survival under hostile conditions, epigenetic modifications, such as DNA methylation, histone post-translational modifications, and non-coding RNAs (ncRNAs)-mediated regulation, suppress proliferative and apoptotic pathways without compromising cell viability. These reversible epigenetic landscapes enable dormant cells to dynamically transition between quiescence and reactivation in response to environmental stimuli [7]. Although surgical resection and systemic therapies can effectively reduce primary tumor burden, recurrence and metastatic progression remain common, especially in patients harboring minimal residual disease (MRD), [8]. Dormant cells that survive initial treatment regimens are thought to be key drivers of relapse and thus represent a compelling therapeutic target [9]. However, the molecular mechanisms that regulate the initiation, maintenance, and reactivation of lung cancer dormancy remain only partially elucidated [10].

Recent studies have identified additional mechanisms contributing to cellular dormancy in lung cancer, including senescence and diapause. Cellular senescence is a state of irreversible cell cycle arrest that can be induced by various stressors, such as DNA damage, oncogene activation, or telomere shortening [11]. In lung cancer, senescent cells can accumulate in response to therapy and contribute to tumor relapse by secreting pro-inflammatory cytokines and growth factors, collectively known as the senescence-associated secretory phenotype (SASP). These factors can promote tumorigenesis, angiogenesis, and immune evasion, thereby facilitating the reawakening of dormant cancer cells [12]. For instance, studies have shown that SASP can influence the tumor microenvironment, creating a niche that supports the survival and reactivation of dormant cells [13].

Diapause, a reversible state of developmental arrest observed in certain organisms, has also been implicated in cancer dormancy [14]. While less studied in the context of lung cancer, diapause-like mechanisms may involve metabolic reprogramming and epigenetic modifications that enable cancer cells to survive under adverse conditions [15]. Research has suggested that certain signaling pathways, such as the mammalian target of rapamycin (mTOR) pathway, play a role in regulating dormancy and may overlap with mechanisms observed in diapause [16].

**Figure 1:**
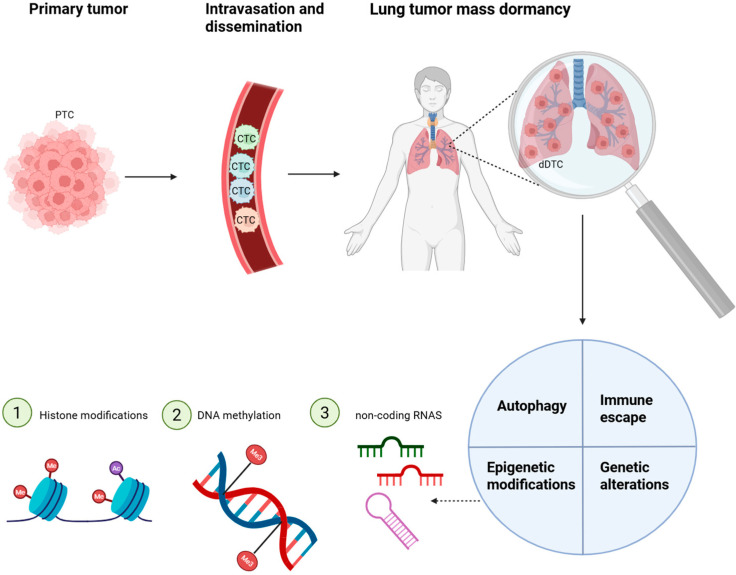
Schematic representation of cancer cell dissemination and lung tumor dormancy regulated by epigenetic and adaptive survival mechanisms. PTCs can intravasate into the bloodstream and circulate as CTCs, eventually disseminating to distant organs such as the lung, where they may enter a dormant state as dDTCs. Within the lung microenvironment, tumor mass dormancy can be maintained by a balance between proliferation and apoptosis. Abbreviations: PTCs, Primary Tumor Cells; CTCs, Circulating Tumor Cells; dDTCs, dormant Disseminated Tumor Cells (Created with BioRender.com, Licensing and Agreement number DP28F9Z1LY, accessed on 23 June 2025).

In this review, the aim is to summarize the latest advances in the biological understanding of dormancy, with a particular focus on epigenetic remodeling and emerging strategies for therapeutically targeting dormant lung cancer cells, aiming to prevent recurrence and achieve durable disease control (Figure 1).

Dormant cancer cells evade therapy and immune detection through main events as well as autophagy, immune escape, genetic alterations, and epigenetic regulation. Particularly, epigenetic control is mediated by coordinated mechanisms such as histone modifications (e.g., methylation and acetylation), DNA methylation, and non-coding RNAs, which could act in synergy with metabolic adaptation via autophagy, immunomodulatory mechanisms facilitating immune escape, and genetic mutations that enhance stress resistance.

## 2. Molecular, Metabolic and Cellular Drivers of Lung Cancer Cell Dormancy

Lung cancer cell dormancy represents a reversible and clinically significant cellular state wherein dormant Disseminated Tumor Cells (dDTCs) undergo proliferative arrest while maintaining long-term viability and the capacity to re-enter the cell cycle, ultimately contributing to disease relapse [17]. Dormant cancer cells are defined by distinct hallmarks, including cell cycle arrest in the G0/G1 phase, therapeutic resistance, reliance on the tumor microenvironmental niche, immune evasion, and the capacity to reactivate cell growth [18].

The molecular and cellular mechanisms underlying tumor dormancy are orchestrated by a complex interplay between intrinsic regulatory pathways, particularly epigenetic modifications, and extrinsic signals derived from the lung tumor microenvironment (TME), including stromal cells, immune components, and extracellular matrix-derived factors.

### 2.1. Molecular Regulation of Dormant Lung Cancer Cells

Tumor dormancy is regulated by a complex interplay of molecular pathways, including autophagy, immune modulation, and angiogenic control. Autophagy, a cellular process responsible for degrading and recycling damaged organelles and proteins, is essential for the survival of dDTCs. By mitigating oxidative stress and maintaining cellular homeostasis, autophagy allows dormant cells to persist under nutrient deprivation and metabolic stress [19]. Key regulators include the Unc-51 Like Autophagy Activating Kinase 1 (ULK1) complex, Activating Molecule in Beclin1-Regulated Autophagy protein 1 (AMBRA1), and Beclin-1, which collectively promote autophagic flux and support cell survival under quiescent conditions [20]. Inhibition of autophagy has been shown to reduce the viability of dormant cells, highlighting its central role in dormancy maintenance [21].

Immune modulation further influences dormancy by balancing tumor elimination and persistence. Cytotoxic T lymphocytes can enforce dormancy through selective elimination of proliferating cells, whereas regulatory T cells (Tregs) and myeloid-derived suppressor cells (MDSCs) create an immunosuppressive environment that allows dormant cells to persist [22]. Dormant cells can evade immune surveillance via mechanisms such as Programmed Death-Ligand 1 (PD-L1) expression, which inhibits T-cell activity, while chronic inflammation or changes in cytokine signaling (e.g., IFN-γ, TNF-α) can trigger reawakening and metastatic outgrowth [23].

Angiogenic regulation is another critical determinant of dormancy. Tumors that fail to induce angiogenesis remain in a dormant state due to limited nutrient and oxygen supply. In lung cancer, the angiogenic switch is governed by the balance between pro-angiogenic factors, such as Vascular Endothelial Growth Factor (VEGF), and anti-angiogenic factors, including thrombospondin-1. Extracellular matrix components further influence endothelial behavior, vessel formation, and the maintenance of dormancy [24]. The integration of these mechanisms highlights the complexity of tumor dormancy and informs therapeutic strategies.

Targeting autophagy may sensitize dormant cells to immune-mediated clearance, while modulation of immune and angiogenic pathways can either maintain dormancy or prevent reawakening [25].

A comprehensive understanding of these interconnected pathways is therefore essential for developing effective epigenetic and combinatorial approaches to prevent metastatic relapse in lung cancer.

### 2.2. Metabolic Modulation of Dormant Tumor States: A Snapshot

Emerging evidence indicates that metabolic reprogramming plays a pivotal role in regulating the epigenetic landscape that sustains cellular dormancy in cancer. Dormant cells frequently downshift glycolysis and rely on oxidative phosphorylation and fatty acid oxidation to maintain energetic balance and redox homeostasis [26]. These metabolic adaptations profoundly influence the availability of key metabolites, such as acetyl-CoA, NAD^+^, α-ketoglutarate (α-KG), succinate, and fumarate, that serve as essential substrates or cofactors for chromatin-modifying enzymes [27]. Reduced glycolytic flux limits acetyl-CoA and thereby histone acetylation, promoting a transcriptionally repressive chromatin state, whereas elevated NAD^+^ levels activate sirtuin deacetylases to reinforce quiescence-associated gene silencing [28]. Similarly, mitochondrial intermediates modulate DNA and histone demethylases of the Ten-eleven translocation (TET) and Jumonji families: high α-KG favors demethylation and potential reactivation of silenced genes, while accumulation of succinate or fumarate stabilizes heterochromatin and sustains dormancy [29].

Lipid metabolism further contributes by generating β-hydroxybutyrate and other short-chain fatty acids that inhibit histone deacetylases, selectively reshaping chromatin accessibility [30].

Together, these metabolic–epigenetic feedback loops allow dormant cancer cells to maintain a low-energy, transcriptionally silent phenotype while retaining the capacity for rapid reactivation upon microenvironmental or therapeutic cues [31].

### 2.3. Targeting Intrinsic Mechanisms of Dormant Cancer Cells in Lung Cancer: A Focus on Epigenetics

Dormant cancer cells possess the capacity to alternate between quiescence and proliferation through dynamic epigenetic reprogramming mechanisms, encompassing DNA methylation, histone modifications, and the regulatory activity of ncRNAs, particularly microRNAs and long ncRNAs, which collectively modulate gene expression networks critical for maintaining dormancy and enabling reactivation under appropriate stimuli [3].

In both non-small-cell lung cancer (NSCLC) and small-cell lung cancer (SCLC) subtypes, increasing levels of DNA methylation are observed in dDTC, predominantly mediated by *DNA methyltransferase 1 (DNMT1)*, [32]. Through this mechanism, methylation patterns are consistently preserved during DNA replication, and the stable silencing of critical cell cycle regulators, as well as cyclin-dependent kinase inhibitor 1A (CDKN1A) and cyclin-dependent kinase inhibitor 2A (CDKN2A), is maintained, thereby leading to G0/G1 phase blockade and the preservation of a dormant phenotype [33,34].

Concurrently, de novo methyltransferases *DNMT3A* and *DNMT3B* show an aberrant hypermethylation of promoter CpG islands at loci involved in cell cycle control, DNA repair, and apoptosis pathways [35]. This includes hypermethylation-mediated silencing of *mutL homolog 1 (MLH1), ataxia-telangiectasia mutated (ATM), and O-6-methylguanine-DNA methyltransferase (MGMT),* which compromises DNA damage response and contributes to chemo-resistance characteristic of dormant lung cancer cells [36,37,38].

In addition, both DNMT3A and DNMT3B extend the epigenetic remodeling of lung cancer cells beyond the classical hypermethylation of promoter CpG islands. These enzymes establish new methylation marks that stably silence tumor suppressor genes, such as CDK and Retinoblastoma 1 (RB1), thereby influencing cell cycle progression and modulating the balance between dormancy and proliferation [1,39]. This de novo methylation contributes to the maintenance of quiescent states in DTCs and supports their adaptive plasticity under microenvironmental or therapeutic stress [40]. Mechanistic insights into the role of DNMT3A and DNMT3B have been obtained using transgenic mouse models. In *KrasG12D-driven* lung cancer models, deletion of Dnmt3a accelerates tumor growth, suggesting that DNMT3A can act as a context-dependent brake on proliferation, potentially reinforcing dormancy in early lesions [41]. Conversely, transgenic mice overexpressing *ΔDNMT3B4-del* specifically in lung epithelial and alveoli cells induces hypermethylation of differentiation-associated loci, enhancing tumorigenic potential and illustrating how aberrant de novo methylation may drive the exit from dormancy and favor tumor progression [42]. These findings highlight that DNMT3A and DNMT3B coordinate broader epigenetic programs in lung cancer cells, controlling cellular plasticity and the potential for reactivation of dormant cells.

Crucially, the DNA methylation pattern in dormant lung cancer cells is dynamic, heterogeneous and context-dependent [43,44]. Focal promoter hypermethylation leads to the silencing of genes involved in proliferative signaling, whereas global hypomethylation of repetitive elements, such as *long interspersed nuclear elements-1 (LINE-1)* and *Alu* sequences, induces genomic instability and enhances epigenetic plasticity, allowing dormant cells to adapt to microenvironmental stressors and maintain long-term survival [44,45]. Moreover, nuclear receptor subfamily 2 group F member 1 (NR2F1) expression has been specifically linked to lung cancer dormancy [46,47]. In lung cancer models, *Sosa MS and colleagues* demonstrated that Nuclear Receptor Subfamily 2 Group F Member 1 (NR2F1) is commonly downregulated via promoter hypermethylation during active tumor proliferation, whereas it is significantly upregulated during cellular dormancy [47]. Furthermore, *NR2F1-driven* dormancy is supported by downstream effectors, including the SRY-Box Transcription Factor 9 (SOX9), retinoic acid receptor β (RARβ), and cyclin-dependent kinase inhibitors, which synergistically enforce cell cycle arrest and promote the long-term quiescence of dormant lung cancer cells [47].

Experimental evidence for these mechanisms has been established using transgenic and genetically engineered mouse models. Conditional overexpression of transcription factors such as NR2F1 or SOX9 in *KrasG12D p53^flox/flox^* NSCLC models promotes long-term quiescence of disseminated tumor cells, demonstrating their roles as dormancy *drivers* in vivo [47]. Reporter systems monitoring the p38/Extracellular signal-regulated kinases (ERK) signaling ratio have allowed the visualization of dormant cell populations within lung and bone marrow niches, showing that high p38 and low ERK activity corresponds to long-lived quiescent cells [48]. These models collectively provide compelling in vivo proof that specific transcriptional and epigenetic programs drive dormancy and can be manipulated to study relapse mechanisms.

Recent mechanistic insights from our group strongly suggest that epigenetic modulation is found to be a central regulator of dormancy-associated signaling via Nuclear factor erythroid 2-related factor 2 (NRF2) and neurogenic locus notch homolog protein (NOTCH) pathways. This supports a model in which promoter hypermethylation of *Kelch-like ECH-associated protein 1 (KEAP1)* and consequent NRF2 activation may establish a survival-oriented, quiescent state through epigenetic reprogramming of *NOTCH1*, thereby facilitating long-term dormancy and resistance to therapy in lung cancer [49,50].

In proliferative states, key dormancy-associated genes are often epigenetically silenced through promoter hypermethylation [51]. However, during dormancy, selective demethylation and chromatin remodeling facilitate the re-expression of these factors, promoting quiescence and long-term survival of dDTCs [52]. Genome-wide methylome analyses further support this model, demonstrating that dormant lung cancer cells exhibit a reversible epigenetic state marked by reversible DNA methylation signatures [53]. Furthermore, dynamic changes in DNA methylation patterns within dormant cells may contribute to phenotypic plasticity, enabling eventual reactivation and metastatic progression [54].

Understanding the interplay between the tumor methylation burden (TMeB) and dormancy-associated epigenetic signatures could provide novel biomarkers for early detection of dormant disease and reveal therapeutic targets to prevent relapse in lung cancer patients [55].

Complementary to DNA methylation, histone modifications modulate chromatin accessibility and gene expression in dormant lung cancer cells [56]. The polycomb repressive complex 2 (PRC2), via its catalytic subunit enhancer of zeste homolog 2 (EZH2), catalyzes the trimethylation of histone H3 on lysine 27 (H3K27me3), leading to transcriptional silencing of differentiation and proliferation-associated genes, thereby reinforcing quiescence and stemness properties essential for dormancy [57,58,59]. In an insightful meta-analysis, *Fan K and collaborators* demonstrated that elevated EZH2 expression correlates with poor prognosis and supports the survival of dormant cells in both NSCLC and SCLC [60].

Concurrently, histone deacetylases (HDACs) repress transcription by removing acetyl groups, while lysine demethylases such as lysine demethylase 5A (KDM5A) and lysine-specific demethylase 1 (LSD1) dynamically modulate histone methylation marks, including H3K4me3 and H3K9me2/3, that correspond to active and repressive chromatin states, respectively [61,62]. Through these modifications, KDM5A and LSD1 are involved in the regulation of gene expression programs that enable dormant lung cancer cells to withstand metabolic stress, hypoxia, and therapeutic pressures [63].

Adding another layer of complexity, specific histone variants, particularly isoforms of macroH2A such as macroH2A1.1 and macroH2A2, have been implicated in the establishment and maintenance of dormancy. These variants replace canonical histones within the nucleosome and influence chromatin structure and gene expression by modulating nucleosome stability and accessibility [64]. Notably, elevated levels of macroH2A isoforms have been linked to the repression of proliferative pathways and the activation of quiescence-associated genes [65].

Moreover, *Sporn JC and colleagues* have previously identified macroH2A variants in dormant lung cancer cells as promising prognostic biomarkers for relapse, highlighting their essential role in maintaining prolonged dormancy [66].

ncRNAs, among which microRNAs (miRNAs), represent an additional layer of intrinsic epigenetic regulation, fine-tuning gene networks implicated in dormancy maintenance and reactivation [67]. These small, ~22-nucleotide RNA molecules regulate target mRNA stability and translation, thereby orchestrating complex regulatory circuits that affect key dormancy-associated pathways [68].

In NSCLC, tumor-suppressive miRNAs such as the miR-29 family are frequently silenced via promoter hypermethylation, resulting in disrupted expression of DNMTs and subsequent aberrant DNA methylation landscapes that reinforce dormancy phenotypes, as reported from *Fabbri M and collaborators* [69]. The loss of miR-29 expression leads to increased DNMT activity, which further silences tumor suppressor genes and genes involved in cell cycle progression, consolidating the quiescent state of dormant disseminated tumor cells. This epigenetic feedback loop illustrates the reciprocal relationship between miRNA regulation and DNA methylation in lung cancer dormancy [70].

Other miRNAs, including miR-199a-5p, modulate metabolic pathways and mitochondrial function, critical for the survival of dormant cells under hypoxic or nutrient-deprived conditions [71]. Furthermore, miRNAs such as miR-200c and miR-205 are pivotal regulators of epithelial-to-mesenchymal transition (EMT), a cellular program linked to enhanced plasticity, invasiveness, and chemoresistance. These miRNAs influence EMT not only through direct targeting of EMT-related transcription factors but also via epigenetic mechanisms involving histone modifications and DNA methylation [72].

By modulating the chromatin state and expression of EMT drivers, these miRNAs contribute to establishing and maintaining a reversible quiescent state that allows cancer cells to evade cytotoxic therapies and immune detection [73]. Furthermore, miRNAs such as miR-708-5p and miR-137 target chromatin remodelers and histone demethylases, maintain G0/G1 cell cycle arrest and suppress proliferative signaling [74,75]. In SCLC, although less extensively characterized, emerging data implicate miRNAs in orchestrating epigenetic reprogramming that supports dormancy and metastatic potential, suggesting conserved mechanisms across lung cancer subtypes [76].

Together, these epigenetic layers, such as DNA methylation, histone modifications, and miRNA-mediated regulation, interact to create a robust but reversible transcriptional landscape that enables lung cancer cells to enter dormancy, evade therapy, and subsequently spread tumor growth [77].

**Figure 2:**
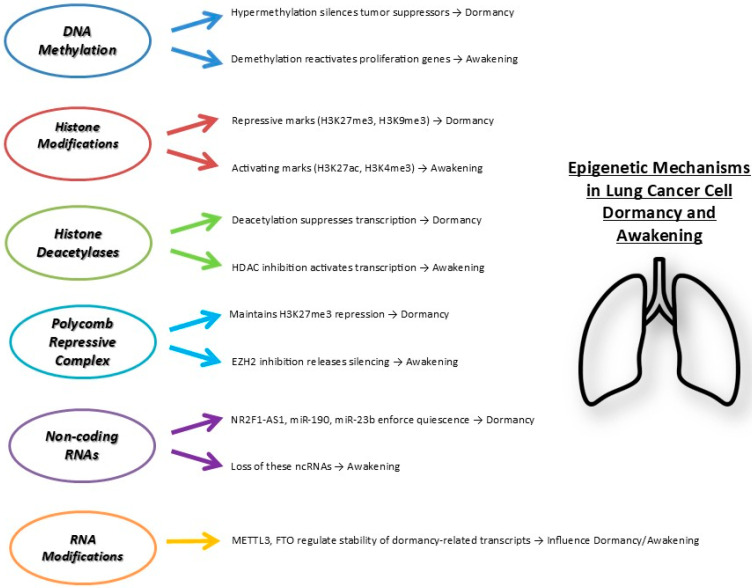
Main epigenetic mechanisms regulating lung cancer cell dormancy and awakening. Multiple layers of epigenetic regulation coordinate the entry into dormancy or reactivation of cancer cells. Repressive marks and factors enforce dormancy, whereas activating marks and loss of inhibitory signals promote cell awakening and proliferation. Abbreviations: H3K27me3, Trimethylation of lysine 27 on histone H3; H3K9me3, Trimethylation of lysine 9 on histone H3; H3K27ac, Acetylation of lysine 27 on histone H3; H3K4me3, Trimethylation of lysine 4 on histone H3. HDACs, Histone Deacetylases; EZH2, Enhancer of Zeste Homolog 2; NR2F1-AS1, Nuclear receptor subfamily 2 group F member 1 antisense RNA 1; miR, microRNA; METTL3, Methyltransferase-like 3; FTO, Fat mass and obesity-associated protein.

Figure 2 summarizes key epigenetic mechanisms controlling lung cancer dormancy.

### 2.4. Modulating Microenvironment-Driven Dormancy in Lung Cancer

Among the key regulators of dormancy, interactions with the extracellular matrix (ECM) via integrins such as α5β1 and αvβ3 activate downstream signaling pathways, including Focal Adhesion Kinase (FAK), Proto-Oncogene Tyrosine-Protein Kinase Src (SRC), and Mitogen-Activated Protein Kinase (MAPK), which collectively promote cytoskeletal organization, survival signaling, and cell cycle arrest, thereby contributing to the maintenance of dormancy in dDTCs within lung tissues [78]. Particularly, integrin-mediated mechanotransduction enables lung cancer cells to sense dynamic changes in ECM composition and mechanical stiffness, including elevated fibrillar collagen density and enhanced crosslinking, which modulate additional downstream effectors such as Phosphoinositide 3-Kinase (PI3K)/protein kinase B (PKB or AKT), Wnt/β-catenin, and Yes-Associated Protein (YAP)/Transcriptional Co-Activator with PDZ-Binding Motif (TAZ) pathways [79]. Furthermore, ECM remodeling through fibronectin assembly, collagen fibrillogenesis, and secretion of matrix remodeling enzymes by activated fibroblasts establishes self-reinforcing feedback loops that sustain the biomechanical and biochemical characteristics of dormancy-supportive niches [80]. Cyclical mechanical strain, such as that generated by normal breathing, and tension exerted by contractile myofibroblasts can also trigger the release of matrix-bound factors, including latent TGFβ, which, upon activation, can further reinforce dormancy signaling or, under pathological remodeling, promote proliferative reawakening [81]. Altogether, the integration of ECM-mediated mechanosensing, downstream survival and quiescence pathways, and epigenetic stabilization positions the ECM as a central regulator of lung cancer cell dormancy, maintaining disseminated tumor cells in a protected, non-proliferative state [82].

Moreover, it is fundamental to understand how ECM components and growth factors in the lung tumor microenvironment may cooperate to regulate DTC dormancy through the modulation of epigenetics. In lung cancer models, culture on laminin, or collagen IV-rich matrices, that mimic the basement membrane environment, attenuates ERK and AKT signaling, and consequently represses invasive gene expression as well as urokinase-type plasminogen activator (uPA) and urokinase plasminogen activator receptor (uPAR), [83]. Concurrently, increased fibrillar collagen density and cross-linking shift the integrin–FAK/Src–p38/ERK balance, generating a low-ERK/high-p38 signaling profile that epigenetically reinforces dormancy [84]. TGFβ and BMP signaling, released from the ECM through mechanical tension or proteolytic remodeling, further sustain dormancy via Suppressor of Mothers Against Decapentaplegic (SMAD)-dependent transcriptional repression [85].

Recent evidence indicates that microenvironmental signals act as dynamic modulators of the epigenetic machinery that sustains quiescence and therapeutic resistance in lung cancer [86]. Cytokines secreted by cancer-associated fibroblasts and macrophages, including Interleukin-6 (IL-6) and Transforming Growth Factor Beta (TGF-β), can activate signal transducer and activator of transcription 3 (STAT3) transcriptional programs that recruit histone modifiers such as EZH2 and HDAC1 to dormancy-associated loci, thereby reinforcing chromatin compaction and the suppression of proliferation-related genes [87,88]. Moreover, mechanical and oxidative stresses transmitted via ECM stiffness or reactive oxygen species can induce ncRNA networks, including miR-101 and long non-coding RNA (lncRNA) HOTAIR, that epigenetically repress proliferation pathways while promoting quiescence and immune evasion [89]. Importantly, dormant tumor cells reciprocally modify their own microenvironment through secretion of lysyl oxidase-like protein 2 (LOXL2), Matrix metalloproteinase-9 (MMP9) and CXC Motif Chemokine Ligand 12 (CXCL12) under epigenetic control, enhancing ECM cross-linking and integrin signaling feedback that stabilizes dormancy niches [90]. Together, these findings delineate a bidirectional axis in which TME-derived signals dynamically remodel tumor epigenomes, while dormant cells epigenetically reprogram their surroundings to maintain a self-reinforcing state of quiescence and persistence.

While some studies have suggested that disruption of integrin-mediated adhesions or inhibition of FAK signaling can sensitize dormant cells to cytotoxic agents [91], accumulating evidence indicates that FAK activity is generally required to sustain dormancy rather than to awaken cells. For example, the early study by *Julio A. Aguirre-Ghiso* [92] demonstrated that suppression of FAK signaling promotes tumor dormancy by limiting ERK activation and favoring p38-mediated growth arrest. More recently, *Liu R and colleagues* [93] provided further support for this concept, showing that low FAK activity correlates with maintenance of dormancy, whereas high FAK signaling promotes proliferation and escape from dormancy.

Additional recent studies further bolster this model, showing that FAK inhibition or decreased FAK phosphorylation is associated with DTC quiescence or dormancy maintenance, while FAK activation or integrin engagement triggers downstream ERK/MAPK pulses that favor cell-cycle re-entry and metastatic outgrowth [94,95,96,97]. Together, these data suggest a complex dual role of integrin–FAK–MAPK axis in dormancy biology: integrins and FAK provide the adhesive-mechanotransductive signals necessary for dormant cell survival in the foreign microenvironment, but a threshold or shift in FAK/MAPK activity may determine exit from dormancy.

The TME’s role extends to modulating immune surveillance, where dormant lung cancer cells evade immune-mediated clearance by upregulating Immune Checkpoint Inhibitors (ICI) such as PD-L1 and downregulating antigen presentation machinery, creating an immunosuppressive niche [98]. This immune evasion is particularly recurrent in SCLC, where rapid shifts in the immune landscape enable dormant cells to persist under immunotherapeutic pressure [99]. Therapeutic strategies aiming to reverse immune evasion, including checkpoint inhibitors targeting Programmed Cell Death Protein 1 (PD-1)/PD-L1 and Cytotoxic T-Lymphocyte-Associated Protein 4 (CTLA-4) pathways, are currently being investigated to eliminate dormant cell reservoirs and prevent relapse [100,101].

Recently, pan-cancer analyses leveraging The Cancer Genome Atlas (TCGA) revealed that global DNA hypomethylation is associated with immune evasion signatures and can influence the effectiveness of anti-PD-1/PD-L1 immunotherapy in lung cancer, even in tumors with high mutational burden [102,103]. It has been demonstrated that PD-L1 hypermethylation constitutes a key mechanism of resistance to anti–PD-L1 therapy. Specifically, methylation at PD-L1 lysine 162 (K162) acts as a negative predictive biomarker for anti–PD-1 treatment in patients with NSCLC and the PD-L1 K162 methylation: PD-L1 ratio provides an even more accurate predictor of therapeutic response [104]. Functionally, PD-L1 DNA methylation influences its expression; although an inverse correlation between methylation and PD-L1 expression is observed in NSCLC cell lines, this relationship is weaker in primary tumor samples [105]. Mechanistically, TGF-β1 can induce PD-L1 promoter demethylation by reducing DNMT1 levels, leading to increased PD-L1 expression [106]. Regarding HDAC, further investigations confirmed its role in the regulation of PD-L1 transcription in lung cancer. HDAC3 is the principal isoform controlling PD-L1 expression: its inhibition increases IFN-γ production and histone acetylation at the PD-L1 promoter, thereby enhancing PD-L1 transcription in tumor cells and elevating PD-L1 levels in dendritic cells within the tumor microenvironment. Additionally, HDAC3 sustains PD-L1 expression in drug-resistant lung cancer cells by repressing histone H3 acetylation at the PD-L1 promoter [107]. In this context, another HDAC family member, HDAC10, has also been positively associated with PD-L1 expression in lung cancer patients [108].

Hypoxia within the TME is another critical extrinsic factor influencing dormancy in lung cancers. Hypoxia-inducible factors (HIFs) orchestrate metabolic reprogramming and the expression of dormancy-associated genes, promoting survival under nutrient-deprived conditions and reinforcing quiescence [109,110]. Targeting hypoxia signaling pathways, for instance, via HIF-1α inhibitors or disruption of angiogenic factors like VEGF, has shown promise in reactivating dormant cells to enhance the efficacy of chemotherapies [111].

Moreover, soluble cytokines and growth factors such as TGF-β, Bone Morphogenetic Proteins (BMPs), and Wingless/Integration-1 signaling pathway ligands (Wnt) derived from stromal fibroblasts and immune cells modulate dormancy through complex paracrine signaling cascades [112]. TGF-β signaling has a dual role, initially suppressing tumor proliferation and later facilitating escape and metastasis, highlighting the therapeutic potential of context-dependent modulation of this pathway [113]. Similarly, Wnt/β-catenin signaling contributes to dormancy regulation by maintaining stemness and promoting resistance phenotypes in NSCLC models [114].

In addition to this, a recent study by *Cuccu A and colleagues* analyzed the transcriptional profiles of quiescent cancer cells derived from xenograft models of NSCLC and other solid cancers, revealing a shared core quiescence-associated program. This conserved signature was characterized by the upregulation of genes linked to stemness, metastatic potential, chemoresistance, and EMT-like traits, including key regulators such as Krüppel-like factor 4 (KLF4) and zinc finger E-box binding homeobox 2 (ZEB2), [115]. Therapeutic targeting of these extrinsic mechanisms requires a nuanced approach given their dynamic and context-dependent nature [116].

Although immune effector cells such as CD8^+^ T lymphocytes can recognize neoantigens expressed by tumor cells and maintain them in a dormant state [117], epigenetic reprogramming in lung cancer cells can profoundly reshape this immune control. CD8^+^ T cells exert antiproliferative effects not only through cytotoxic activity but also via the secretion of cytokines such as IFN-γ, TNF-α, and lymphotoxin. Among these, IFN-γ acts as a key mediator of dormancy by activating STAT1-dependent transcription of cell cycle inhibitors p21^WAF1/CIP1^ and p27^Kip1^ [118] and by indirectly suppressing angiogenesis and enhancing antitumor immunity [119]. Th1 cells contribute similarly by producing cytokines that limit tumor proliferation [120]. Moreover, both CD4^+^ and CD8^+^ T cells have been shown to restrain late metastatic outgrowth in murine models of chemical carcinogenesis, indicating that adaptive immune surveillance is crucial in sustaining dormancy [121].

Recent studies highlight that epigenetic modifications, particularly DNA methylation and histone marks, intersect with the TME to regulate dormancy and immune evasion in lung cancer [122]. In NSCLC, *Ke X and colleagues* demonstrated that tumor cells might induce demethylation of the *forkhead box P3 (FOXP3)* promoter in CD4+ T cells, leading to an increased number and activity of Treg cells. These Tregs secrete immunosuppressive cytokines, including Interleukin-10 (IL-10) and TGF-β1, which dampen the proliferation and activity of effector T cells. This epigenetically driven immunosuppressive microenvironment reduces immune surveillance, creating conditions that allow dDTCs to survive, evade clearance, and potentially drive late relapse or metastatic progression [123].

In addition, *Li and colleagues* showed that epigenetic modifications influence angiogenesis and TME regulation, with DNA methylation patterns correlating with differential patient outcomes following chemotherapy combined with bevacizumab [124]. It has recently been reported that, under hypoxic conditions, HIF-1α regulates DNA methylation enzymes—ten-eleven translocase-2 (TET2) and DNMT3a—to control S100 calcium-binding protein A6 (S100A6) transcription in lung cancer cells.

Hypoxia-induced CpG hypomethylation at the *S100A6* promoter enhances its expression, promoting proliferation, migration, and metastasis. HIF-1α-dependent activation of S100A6 involves TET2 enrichment and reduced DNMT3a binding, linking epigenetic remodeling to tumor progression. Functionally, this pathway may influence tumor dormancy and TME by creating hypoxia-driven niches that support the survival of DTCs and facilitate immune evasion [125].

In parallel, histone modifications critically influence immune evasion programs. Histone demethylase jumonji domain containing protein 2C (JMJD2C), which interacts with HIF-1α, removes repressive H3K9me3 marks, thereby activating the transcription of target genes, which not only promote metastatic lung cancer but may also facilitate the exit of dormant disseminated tumor cells from quiescence, linking hypoxia-driven epigenetic remodeling to both metastasis and dormancy reactivation [126].

Emerging strategies integrate inhibition of ECM–receptor interactions, ICI blockade, and modulation of hypoxia and paracrine signaling, often in combinatorial regimens, to disrupt the supportive microenvironment and eradicate dormant lung cancer cells [127,128].

Such multi-modal interventions hold promise to prevent tumor relapses and improve long-term outcomes in lung cancer patients.

## 3. Therapeutic Advances in Overcoming Lung Cancer Cell Dormancy

**Figure 3:**
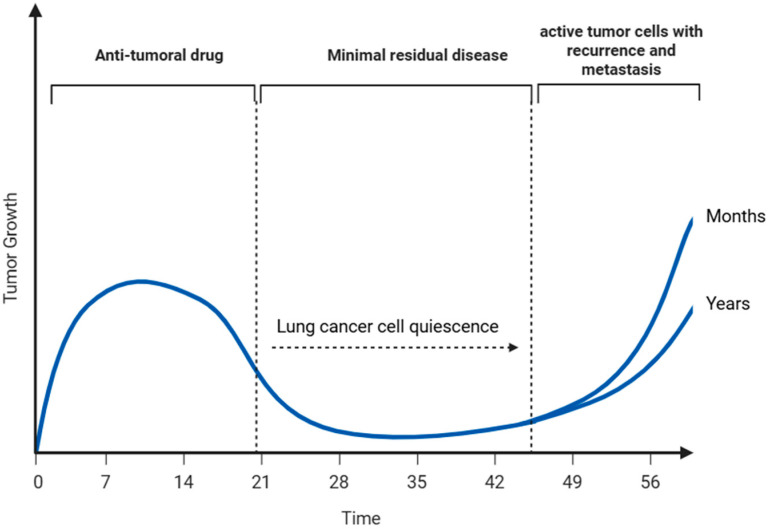
Quiescent lung cancer cells (QLCCs) as a reservoir for tumor recurrence and metastasis. These QLCCs may persist at the primary tumor site or disseminate through the vasculature to distant tissues. Over time, and under specific microenvironmental or systemic cues, QLCCs can re-enter the cell cycle in an active state, driving tumor relapse and metastatic outgrowth after prolonged periods of clinical dormancy lasting months or even years (Created with BioRender.com, Licensing and Agreement number VG28FKCXGG, accessed on 25 June 2025).

Although lung cancer treatments have advanced considerably, achieving sustained disease control continues to be a challenge for many patients. The failure to eliminate residual dormant tumor cells after treatment remains a key factor underlying recurrence and metastatic spread in lung cancer patients [129,130]. As illustrated in Figure 3, although most proliferative lung cancer cells are effectively eliminated by conventional treatments, a subpopulation of quiescent lung cancer cells (QLCCs) evade eradication by entering a reversible G0 phase, maintaining viability either at the primary tumor site or at distant metastatic niches. This dormant state, regulated by complex microenvironmental and systemic cues, allows these cells to persist undetected for extended periods before re-entering the cell cycle to drive tumor relapse and metastatic outgrowth [131].

Addressing this challenge requires therapeutic strategies that either maintain dormancy (“sleeping strategy”), forcibly reactivate dormant cells (“awakening strategy”), or selectively eliminate them (“killing strategy”), (Table 1). Current therapeutic advances have largely focused on targeting extrinsic signaling pathways that govern tumor dormancy [132,133,134]. For example, inhibition of integrin-mediated adhesion signaling has demonstrated efficacy in reducing dormant cell survival and preventing reactivation, and several of these approaches have advanced into clinical trials in NSCLC patients, often in combination with standard chemotherapy or radio-chemotherapy regimens [135].

Within the “sleeping strategy,” induction of the nuclear receptor NR2F1 through epigenetic modulators such as 5-aza-2′-deoxycytidine (5-Aza-C), alone or in combination with all-trans retinoic acid (ATRA), represents a prototypical example of reinforcing dormancy to prevent metastatic relapse [47]. However, given the dual epigenetic and cytotoxic effects of 5-Aza-C, its classification as purely a “sleeping” agent is sometimes ambiguous. A more selective alternative is the NR2F1 agonist C26, which has been validated in preclinical models as a non-cytotoxic inducer of dormancy. C26 promotes a NR2F1^hi^/p27^hi^/Ki-67^lo^ phenotype, enforces growth arrest, and suppresses metastatic outgrowth without inducing apoptosis, providing a clear mechanistic example of a “sleeping strategy” that specifically targets dormancy-associated transcriptional programs [136].

Additional approaches include pharmacologic modulation of the TGF-β pathway, which reinforces quiescence [113], and inhibition of the Wnt/β-catenin pathway, which helps stabilize dormancy states [114]. Beyond NR2F1 induction, a broader range of epigenetic interventions is emerging as highly relevant. Inhibitors of histone methyltransferases and demethylases such as EZH2, KDM5A, and LSD1 have shown the ability to reprogram drug-tolerant states or impair survival of dormant cancer cells, with several agents (e.g., tazemetostat, CPI-455, ORY-1001, GSK2879552) already in preclinical development or early-phase clinical evaluation [137,138,139]. These epigenetic modulators exemplify a mechanistically coherent “sleeping” strategy by reinforcing a transcriptionally repressive state that restricts tumor regrowth, while also providing opportunities for combination therapies.

Conversely, “awakening strategies” seek to sensitize dormant cells to cytotoxic agents by forcing them into active proliferation. For example, *Cho and collaborators* demonstrated that phosphodiesterase 5 (PDE5) inhibitors, when combined with chemotherapy, can trigger re-entry into the cell cycle and thereby increase susceptibility to conventional treatments [140]. Importantly, epigenetic reprogramming may also serve as an awakening strategy: preclinical evidence indicates that combined inhibition of EZH2 and HDACs can overcome epigenetically mediated drug tolerance, restoring sensitivity to standard therapies [141]. Other awakening strategies include targeting hypoxia pathways with HIF-1α inhibitors to disrupt survival niches [142] and modulating angiogenesis through VEGF inhibition to reactivate quiescent cells [143].

Finally, “killing strategies” aim to directly eradicate dormant cancer cells through the simultaneous inhibition of survival pathways and chemotherapy. Combination regimens involving cytotoxic agents with inhibitors of Src kinase or cyclooxygenase-2 (COX-2) have shown promise in selectively targeting dormant subpopulations [144]. In addition, epigenetic targeting of LSD1 has been reported to impair the persistence of drug-tolerant persister cells, supporting its integration into “killing” approaches [139]. Immunotherapeutic interventions also hold potential: immune checkpoint inhibitors targeting PD-1/PD-L1 or CTLA-4 can promote immune clearance of dormant tumor cells [145], while Chimeric Antigen Receptor (CAR) T cell or Natural Killer (NK)-based therapies directed against dormancy-associated antigens represent emerging strategies for selective eradication [146].

Together, these examples highlight that while modulation of extrinsic pathways remains crucial, incorporating epigenetic modulators into the conceptual framework of “sleeping,” “awakening,” and “killing” strategies provides a more mechanistically consistent and translationally relevant roadmap for therapeutic interventions against lung cancer dormancy.

**Table 1:** Therapeutic strategies for dormant cancer cells: from maintaining dormancy (“Sleeping”), to reactivating cells to enhance treatment sensitivity (“Awakening”) and eliminating cells via combined therapies (“Killing”).

Approach	Treatment Modality	Refs.
**Sleeping strategy**	Inhibition of integrin-mediated signaling pathways (e.g., α5β1, αvβ3 integrins; FAK/SRC inhibitors)	[133,134]
	Induction of NR2F1 by 5-azacytidine (5-Aza-C) alone or in combination with all-trans-retinoic acid (ATRA)	[47]
	Epigenetic modulation: inhibitors targeting histone methyltransferases/demethylases such as EZH2, KDM5A, LSD1	[137,138,139]
	NR2F1 agonist C26, a selective non-cytotoxic dormancy inducer	[136]
	TGF-β pathway modulators to reinforce quiescence	[147]
	Wnt/β-catenin pathway inhibitors to stabilize dormancy	[148]
**Awakening strategy**	PDE5 inhibitors in combination with chemotherapy to sensitize dormant cells	[140]
	Combined EZH2 and HDAC inhibition to epigenetically reprogram dormant cells	[141]
	Targeting hypoxia pathways (HIF-1α inhibitors) to disrupt survival niches	[142]
	Modulation of angiogenesis (VEGF inhibitors) to reactivate quiescent cells	[143]
**Killing strategy**	Chemotherapy combined with Src or COX-2 inhibitors to eliminate dormant cells	[144]
	LSD1 inhibition to eradicate drug-tolerant persister cells	[139]
	Immune checkpoint inhibitors (PD-1/PD-L1, CTLA-4) to promote clearance of dormant cells	[145]
	CAR-T or NK cell–based therapies targeting dormancy-associated antigens	[146]

Abbreviations: 5-Aza-C, 5-Azacytidine; ATRA, All-Trans-Retinoic Acid; NR2F1, Nuclear Receptor Subfamily 2, Group F, Member 1; EZH2, Enhancer of Zeste Homolog 2; KDM5A, Lysine Demethylase 5A; LSD1, Lysine-Specific Demethylase 1; FAK, Focal Adhesion Kinase; SRC, Proto-Oncogene Tyrosine-Protein Kinase Src; TGF-β, Transforming Growth Factor Beta; Wnt, Wingless/Integration-1 signaling pathway; PDE5, Phosphodiesterase Type 5; HDAC, Histone Deacetylase; HIF-1α, Hypoxia-Inducible Factor 1 Alpha; VEGF, Vascular Endothelial Growth Factor; PD-1, Programmed Cell Death Protein 1; PD-L1, Programmed Death-Ligand 1; CTLA-4, Cytotoxic T-Lymphocyte-Associated Protein 4; CAR-T, Chimeric Antigen Receptor T-cell; NK, Natural Killer.

Several molecular targets have emerged as critical nodes in dormancy regulation, many of which are under clinical evaluation (Table 2), [149]. Firstly, agents such as volociximab, a monoclonal antibody targeting integrin α5β1, and cilengitide, a cyclic peptide targeting integrin αvβ3, have shown potential in disrupting integrin-mediated signaling pathways that support dormant cancer cell survival [150,151].

Consequently, the use of FAK inhibitors such as GSK2256098 and defactinib (VS-6063) in clinical trials—either as monotherapy or in combination with immunotherapies—likely functions by disrupting tumor–microenvironment interactions that are critical for survival but do not inherently “awaken” dormant cells. Therefore, FAK blockade should be considered in the context of combination strategies where dormant cell sensitization is required, rather than as a direct inducer of cell cycle re-entry. This clarification aligns preclinical and clinical observations and highlights that the effect of FAK inhibition on dormancy is context-dependent, depending on the microenvironmental cues and co-administered therapies [152,153,154].

Similarly, targeting inflammatory and signaling mediators such as Janus kinases (JAK1/2) with ruxolitinib or pacritinib in *EGFR-mutant* NSCLC [155,156] and STAT3 with OPB-51602, offers promising avenues to overcome therapy resistance associated with dormant cells [157]. *Edelman MJ and coworkers* reported that COX inhibitors, such as celecoxib, have reached phase III trials [158], aiming to impair inflammatory pathways that support tumor cell survival during dormancy.

Beyond these approaches, epigenetic regulators have also emerged as therapeutic targets in dormancy modulation. Inhibitors of histone-modifying enzymes (EZH2, KDM5A and LSD1), are under evaluation at different stages of development. For example, the EZH2 inhibitor tazemetostat is currently in phase II trials (NCT05023655) as monotherapy or in combination with immune checkpoint blockade in solid cancers, including lung cancer [159]. KDM5A inhibition with CPI-455 has shown preclinical efficacy in reversing epigenetic reprogramming in NSCLC drug-tolerant cells [138]. Similarly, LSD1 inhibitors such as ORY-1001 (preclinical), tested alone or in combination with γ-secretase inhibitors (DBZ and RO4929097) in SCLC patient-derived xenograft models, and GSK2879552 (phase I, NCT02034123) in relapsed/refractory SCLC, highlight the potential of targeting chromatin modifiers to impair dormancy-associated survival programs [160,161]. Moreover, combined blockade of HDAC and EZH2 are being explored to achieve stronger epigenetic reprogramming in NSCLC cells [141], (Table 2).

Collectively, these therapeutic interventions reflect a multipronged approach targeting the unique biology of dormant lung cancer cells. By integrating strategies to maintain, awaken, or eradicate dormant populations, ongoing clinical efforts hold promise for reducing late relapse and improving long-term outcomes in lung cancer patients.

To provide a concise overview of the current therapeutic landscape for targeting tumor dormancy, Table 3 summarizes the main strategies, their underlying mechanisms, and the respective advantages and limitations. These approaches range from epigenetic modulators that reactivate or silence dormancy-related genes to autophagy inhibitors that impair survival pathways, immune checkpoint inhibitors that restore anti-tumor immunity, and anti-angiogenic therapies that maintain vascular dormancy. By outlining both the potential benefits and inherent challenges of each approach, this table provides a framework to guide future research and clinical translation in the management of dormant tumor cells.

### Epigenetic Modifiers as Emerging Tools in the Management of Lung Cancer Cell Dormancy

Pharmacologic epigenetic reprogramming offers two mechanistically distinct approaches to eradicate or control disseminated dormant NSCLC cells: (a) epigenetic priming/wake-and-kill, where low-dose DNMTis and/or HDACis reverse promoter hypermethylation and repressive chromatin to re-express tumor-suppressor, antigen-presentation and interferon-stimulated genes, thereby converting “immune-cold” dormant cells into more immunogenic and chemosensitive targets [168], and (b) epigenetic enforcement of dormancy, where activation of transcriptional repressors such as NR2F1 establishes a durable quiescence program that blocks metastatic outgrowth but may require long-term maintenance [136].

Preclinical and translational studies have shown that sequential low-dose azacitidine or decitabine can induce viral-mimicry via dsRNA/IFN signaling and upregulation of Major Histocompatibility Complex (MHC), thereby reducing immune evasion and sensitizing NSCLC to chemotherapy or immunotherapy [169]. Targeting histone modifiers offers complementary benefits: inhibition of EZH2, the catalytic subunit of PRC2, reverses H3K27me3-mediated repression of differentiation and anti-proliferative programs, reduces metastatic colonization capacity, and can synergize with immune activation by derepressing interferon-responsive loci [170].

HDAC inhibitors such as entinostat and vorinostat reconfigure acetylation landscapes to reactivate cell-cycle and apoptotic networks suppressed in dormant cells, and potentiate DNMTi or cytotoxic effects in NSCLC models as well as in early clinical trials [171]. Nevertheless, their single-agent activity in solid tumors remains limited and requires rational scheduling to avoid paradoxical induction of cellular plasticity. A complementary RNA-centered axis further underscores the dual paradigm of “enforce-dormancy” versus “wake-and-kill”: NR2F1 and its regulatory long non-coding RNA NR2F1-AS1 mediate a broad repressive chromatin program; pharmacologic NR2F1 agonists or lncRNA perturbation can either maintain dormancy (preventing overt metastasis) or generate vulnerabilities when combined with cytotoxic or immune strategies [10,136].

Finally, contemporary translational work supports rational combinations (DNMTi ± HDACi ± EZH2i → immunotherapy or cytotoxin) for minimal-residual NSCLC, but mandates: (a) dormancy-specific biomarkers as well as circulating DTC, (b) patient selection based on tumor epigenomic/immunologic status, and (c) tightly controlled dosing/sequencing to limit off-target gene reactivation (including potential oncogene derepression) and to maximize induction of viral-mimicry/antigenicity rather than adverse plasticity. Early-phase trials and correlative studies cited above provide the mechanistic and clinical rationale, but larger, biomarker-guided studies are required to define safety, optimal sequence and efficacy specifically for targeting dormant NSCLC cells [172].

## 4. Clinical Recognition of Tumor Dormancy in Lung Cancer

Tumor dormancy in lung cancer is a clinically significant phenomenon characterized by a prolonged period of disease-free survival (DFS) following initial treatment, during which DTCs remain viable but quiescent. These dormant cells can evade detection through conventional imaging and may later reawaken, leading to late recurrence [173].

Understanding the clinical manifestations and challenges associated with tumor dormancy is crucial for improving patient outcomes. Patients with lung cancer may experience extended periods of remission, only to present with recurrent disease years after initial treatment. This late recurrence often occurs in the form of isolated metastases, commonly in the brain, bone, or adrenal glands [174]. The detection of dormant tumor cells poses significant challenges due to their quiescent nature and the limitations of current imaging modalities. Standard imaging techniques, such as computed tomography (CT) and positron emission tomography (PET), may not identify dormant cells, leading to false-negative results [175].

Moreover, the absence of symptoms during the dormant phase further complicates early detection. Advances in liquid biopsy techniques, including the detection of circulating tumor cells (CTCs) and tumor-derived extracellular vesicles (EVs), offer promising avenues for monitoring MRD and identifying dormant cells. These methods have the potential to provide real-time insights into the presence of dormant tumor cells and their reactivation [176,177]. Retrospective studies have documented instances of late recurrence in lung cancer patients, emphasizing the clinical significance of tumor dormancy. For example, a study analyzing recurrence patterns in NSCLC patients found that a subset of patients experienced recurrence more than five years after initial treatment. These findings suggest that dormant tumor cells can persist for extended periods before reactivating and causing relapse [178].

Importantly, certain subsets of drug-tolerant cancer cells, often referred to as persister cells, enter a quiescent state that allows them to evade cytotoxic effects of chemotherapy and act as a reservoir for therapy-resistant clones, particularly under targeted treatments [179]. These cells frequently share characteristics with cancer stem cells, including self-renewal capacity, cellular plasticity, tumor-initiating potential, and prolonged dormancy, highlighting the critical need to understand how chemotherapy influences stemness and dormancy programs [180]. Chemotherapy paradoxically reinforces dormancy by selectively eliminating proliferating cells while sparing quiescent DTCs that retain pre-established epigenetic states. These states include DNA hypermethylation at promoters of proliferation-associated genes and histone deacetylation via HDACs, collectively maintaining G0/G1 arrest [181]. This is in line with recent studies that suggested how epigenetic mechanisms, including aberrant DNA methylation, play a key role in maintaining disseminated tumor cells in a prolonged dormant state while preserving their capacity for future proliferation [182]. Exposure to chemotherapeutic stress, such as DNA damage and oxidative stress, further engages stress–response pathways, as previously reported (i.e., p38, MAPK), which recruit chromatin-modifying enzymes (DNA methyltransferases, histone methyltransferases, HDACs, and demethylases) to dormancy-related loci. This leads to reinforced expression of cell cycle inhibitors such as CDKN1A/p21 and CDKN2A/p16, sustained suppression of proliferation and apoptosis, and stabilization of the dormant phenotype [183,184]. Through these epigenetic mechanisms, chemotherapy unintentionally consolidates quiescence, creating a population of DTCs that is resistant to treatment and primed for eventual metastatic relapse, although the underlying mechanisms remain to be fully elucidated [185].

Under chemotherapeutic stress, *Wang L and colleagues* demonstrated that cisplatin profoundly alters the splicing landscape of NSCLC cells. Integration of PacBio long-read isoform sequencing with short-read next-generation sequencing enabled the identification of aberrant splicing events and transcriptome-wide isoform shifts. When compared with noncancerous counterparts as well as dormant and reactivated cell states, cisplatin exposure was associated with a marked contraction of isoform diversity, largely attributable to disrupted exon inclusion/exclusion dynamics [186].

Moreover, *Carlson P and team* provided critical insights into the mechanisms of chemoresistance in DTCs. In fact, the researchers demonstrated that resistance was not attributable to cell cycle arrest but was instead driven by integrin-mediated interactions with the ECM. Dormant DTCs displayed elevated integrin expression, which functionally shielded them from chemotherapy-induced cytotoxicity by enhancing adhesion-dependent survival signaling. Importantly, pharmacologic inhibition of integrins abrogated this protective effect, thereby restoring sensitivity of dormant cells to chemotherapeutic agents [187]. Understanding the patterns of late recurrence, the challenges in detecting dormant cells, and the insights gained from case studies and retrospective analyses can inform strategies for monitoring and treating patients at risk of relapse [188,189].

Incorporating these considerations into clinical practice may enhance the ability to identify and manage dormant tumor cells, ultimately leading to more effective interventions and improved survival rates.

## 5. Conclusions

Dormancy in lung cancer represents a major clinical challenge, contributing to therapeutic resistance, relapse, and metastatic outgrowth. The reversible quiescent state allows cancer cells to evade immune surveillance and cytotoxic therapies [3].

This review highlights the following key points: (1) dormancy is maintained by a dynamic interplay between intrinsic (e.g., genetic mutations, epigenetic reprogramming) and extrinsic (e.g., microenvironmental stress, immune modulation) factors [52]; (2) epigenetic mechanisms, including DNA methylation, histone modifications, and ncRNAs, play pivotal roles in repressing proliferation and apoptosis pathways, thus preserving viability during dormancy [51,190]; (3) dormant cancer cells exhibit high plasticity, enabling transitions between dormancy and reactivation in response to environmental cues, and contributing to MRD and recurrence [191]; (4) despite growing knowledge, the molecular underpinnings of dormancy induction, maintenance, and reawakening remain incompletely understood, particularly in lung cancer [1]; (5) targeting the epigenetic landscape of dormant cells offers promising therapeutic avenues to eliminate residual disease and prevent relapse, especially when combined with existing or novel agents [192]; (6) comprehensive multi-omics and functional analyses are essential to identify dormancy-specific epigenomic patterns and to establish robust biomarkers for predicting disease progression [193].

Overall, elucidating the epigenetic mechanisms underlying cellular dormancy offers a pivotal foundation for the development of precision therapies designed to eliminate dormant cancer cells, improve durable therapeutic responses, and ultimately transform long-term clinical management and outcomes in lung cancer care.

## Figures and Tables

**Table 2:** Key molecular targets and corresponding therapeutic agents are currently under preclinical and clinical investigations for reactivating dormant lung cancer cells.

Molecular Target	Drug/Agent	Phase of Clinical Study	Treatment Type	Cancer Type	Ref.
**Integrin α5β1**	Volociximab	Phase Ib (NCT00666692, NCT00654758)	Combinatory (carboplatin, paclitaxel, bevacizumab)	Advanced NSCLC	[150]
**Integrin αvβ3**	Cilengitide	Phase I (NCT01118676)	Combinatory (with radiochemotherapy)	Locally advanced NSCLC	[151]
**FAK**	GSK2256098	Phase II (NCT01951690)	Single	*KRAS-mutant* NSCLC	[152]
		Phase I (NCT03875820)	Combinatory (with RO5126766)	NSCLC and other solid tumors (low-grade serous ovarian, colorectal cancer)	[153]
	VS-6063	Phase I/II (NCT02758587)	Combinatory (with pembrolizumab)	NSCLC and other solid tumors (mesothelioma, pancreatic neoplasm)	[154]
**JAK2**	Pacritinib	Phase I (NCT02342353)	Combinatory (with erlotinib)	*EGFR-mutant* NSCLC	[155]
**JAK1/2**	Ruxolitinib	Phase I/II (NCT02155465)	Combinatory (with erlotinib)	*EGFR-mutant* lung adenocarcinoma with acquired resistance to erlotinib	[156]
**STAT3**	OPB-51602	Phase I (NCT01184807)	Single	Advanced solid tumors, particularly NSCLC	[157]
**COX1/2**	Celecoxib	Phase III (NCT01041781)	Combinatory (gemcitabine, pemetrexed disodium, carboplatin)	Advanced NSCLC	[158]
**EZH2**	Tazemetostat	Phase II (NCT05023655)	Single/Combination with ICB	Solid cancers, inclunding lung cancer	[159]
**KDM5A**	CPI-455	Preclinical	Epigenetic reprogramming	NSCLC drug-tolerant cells	[138]
**LSD1**	ORY-1001	Preclinical	Single or in combination with two GSIs, DBZ and RO4929097	SCLC PDX models	[160]
	GSK2879552	Phase I (NCT02034123)	Single	Relapsed/Refractory SCLC	[161]
**HDAC/EZH2**	Dual inhibition	Preclinical	3-deazaneplanocin A plus HDAC inhibitor vorinostat	In vitro NSCLC cells	[141]

Abbreviations: NSCLC, Non-Small Cell Lung Cancer; SCLC, Small Cell Lung Cancer; FAK, Focal Adhesion Kinase; JAK, Janus Kinase; STAT3, Signal Transducer and Activator of Transcription 3; COX, Cyclooxygenase; EZH2, Enhancer of Zeste Homolog 2; KDM5A, Lysine Demethylase 5A; LSD1, Lysine-Specific Demethylase 1; HDAC, Histone Deacetylase; PDX, Patient-Derived Xenograft; ICB, Immune Checkpoint Blockade; KRAS, Kirsten Rat Sarcoma Viral Oncogene Homolog; EGFR, Epidermal Growth Factor Receptor.

**Table 3:** Advantages and Limitations of Current Therapeutic Strategies Targeting Tumor Dormancy.

Strategy	Mechanism	Advantages	Limitations	Ref.
**Epigenetic Modulators**	Reactivation or silencing of dormancy-related genes (e.g., via DNMT or HDAC inhibitors)	Potential to reverse dormancy-associated silencing; clinically available agents	Non-specific effects; possible reactivation of oncogenes; limited data in dormancy-specific context	[162]
**Autophagy Modulation**	Inhibition of autophagy to prevent survival of dormant cells	Targets a key dormancy survival mechanism	Risk of toxicity; autophagy may play dual roles (pro- and anti-tumor)	[163]
**Immune Checkpoint Inhibitors**	Reactivation of anti-tumor immunity	Effective in reactivating immune surveillance; clinically validated agents	Limited efficacy in dormant tumors due to low immunogenicity or immune evasion	[164]
**Anti-Angiogenic Therapy**	Maintenance of angiogenic dormancy via VEGF inhibition	Can delay relapse by limiting vascular support	May promote resistance or increased invasiveness post-therapy	[165]
**Pro-Dormancy Induction**	Use of agents like NR2F1 agonists to keep cells in a dormant state	May prevent recurrence by maintaining dormancy	Long-term dormancy risk; requires continuous monitoring; limited clinical evidence	[97]
**Cell Cycle Inhibitors (e.g., CDK4/6)**	Forces exit from quiescence to sensitize to chemotherapy	Enhances response to cytotoxic agents	May awaken dormant cells, leading to rapid proliferation if not properly controlled	[166]
**Combination Therapies**	Dual targeting (e.g., epigenetics + immunotherapy)	Synergistic effects may overcome monotherapy resistance	Increased complexity; higher risk of adverse events	[167]

Abbreviations: DNMT, DNA Methyltransferase; HDAC, Histone Deacetylase; NR2F1, Nuclear Receptor Subfamily 2 Group F Member 1; VEGF, Vascular Endothelial Growth Factor; CDK, Cyclin-Dependent Kinase.

## Data Availability

No new data were created or analyzed in this study. Data sharing is not applicable to this article.
